# *Staphylococcus aureus* Promotes Smed-PGRP-2/Smed-setd8-1 Methyltransferase Signalling in Planarian Neoblasts to Sensitize Anti-bacterial Gene Responses During Re-infection

**DOI:** 10.1016/j.ebiom.2017.04.031

**Published:** 2017-04-24

**Authors:** Cedric Torre, Prasad Abnave, Landry Laure Tsoumtsa, Giovanna Mottola, Catherine Lepolard, Virginie Trouplin, Gregory Gimenez, Julie Desrousseaux, Stephanie Gempp, Anthony Levasseur, Laetitia Padovani, Emmanuel Lemichez, Eric Ghigo

**Affiliations:** a CNRS UMR 7278, IRD198, INSERM U1095, APHM, Institut Hospitalier Universitaire Méditerranée-Infection, Aix-Marseille Université, 19-21 Bd Jean Moulin 13385, Marseille, Cedex 05, France; b UMR MD2, Faculté de Médecine Nord, Aix Marseille University and Institute of Research in Biology of the French Army Marseille, France; c Laboratory of Biochemistry, La Timone University Hospital, Assistance Publique Hôpitaux de Marseille, Marseille, France; d Otago Genomics & Bioinformatics Facility, Department of Biochemistry, University of Otago, PO Box 56, 710 Cumberland Street, Dunedin 9054, New Zealand; e APHM, Timone Hospital, Department of Radiotherapy, Marseille 13005, France; f UCA, Inserm, U1065, C3M, Université de Nice Sophia-Antipolis, Equipe labellisée Ligue Contre le Cancer, 06204 Nice Cedex 3, France

**Keywords:** Neoblasts, Planarians, *S. aureus*, PGRP-2, sted8-1, Instructed immunity, Stem cells

## Abstract

Little is known about how organisms exposed to recurrent infections adapt their innate immune responses. Here, we report that planarians display a form of instructed immunity to primo-infection by *Staphylococcus aureus* that consists of a transient state of heightened resistance to re-infection that persists for approximately 30 days after primo-infection. We established the involvement of stem cell-like neoblasts in this instructed immunity using the complementary approaches of RNA-interference-mediated cell depletion and tissue grafting-mediated gain of function. Mechanistically, primo-infection leads to expression of the peptidoglycan receptor *Smed-PGRP-2*, which in turn promotes *Smed-setd8-1* histone methyltransferase expression and increases levels of lysine methylation in neoblasts. Depletion of neoblasts did not affect *S. aureus* clearance in primo-infection but, in re-infection, abrogated the heightened elimination of bacteria and reduced *Smed-PGRP-2* and *Smed-setd8-1* expression. *Smed-PGRP-2* and *Smed-setd8-1* sensitize animals to heightened expression of *Smed-p38 MAPK* and *Smed-morn2*, which are downstream components of anti-bacterial responses. Our study reveals a central role of neoblasts in innate immunity against *S. aureus* to establish a resistance state facilitating *Smed-sted8-1*-dependent expression of anti-bacterial genes during re-infection.

## Highlights

• Planarians initiate a genetic program of instructed immunity during *S. aureus* infection.
• Planarians neoblasts have a critical function in controlling the heightened expression of *Smed-PGRP-2* and *Smed-setd8*-1.
• Instructed immunity can be grafted onto naive animals.

Research in context

Little is known about how organisms exposed to recurrent infections adapt their innate immune responses. Most studies addressing this question in vertebrates have been performed on immune cells which are already trained for immune function. We established that planarians are endowed with instructed immunity allowing them to clear *S. aureus* with a higher efficacy during re-infection. We define the central role of neoblasts and Smed-PGRP-2 for establishing a resistance state against *S. aureus* that is controlled by Smed-sted8 for facilitated expression of anti-bacterial genes during re-infection. This shed light on the role of stem cells and epigenetic determinant in controlling innate immune memory.

## 1 Introduction

*Staphylococcus aureus* persistently colonizes the skin and mucosa of 20% of the human population and is a major cause of severe infections (Lowy, 1998; Edwards and Massey, 2011). There has been a dramatic increase in antibiotic-resistant *S. aureus* linked to chronic infections, emphasizing the importance of characterizing the molecular basis of the immune mechanisms involved in re-infection. Vertebrate immunity relies on a first-line innate immune defence of low specificity that induces parallel highly specific adaptive responses that persist over long periods of time as a type of immune memory (Delves et al., 2006, Murphy et al., 2014, Moret and Siva-Jothy, 2003). Vertebrates, such as mice, that lack B and T cells are protected against secondary infections after primo-infection or vaccination (Bistoni et al., 1986, 1988). A few studies have revealed the existence of different forms of innate immune memory in vertebrates and invertebrates, defined as heightened innate immune responses, against previously encountered pathogens (Netea et al., 2016, Milutinovic and Kurtz, 2016). This so-called trained immunity has attracted much attention given its potential as a strategy to promote immune resistance against pathogens. To date, several ways have been defined to use the trained immunity concept as novel therapeutic approach to fight infectious diseases. Vaccines which combine adaptative immune memory and trained immunity, or the use of inducer of trained immunity as vaccine adjuvant (Netea et al., 2016).

Trained immunity in vertebrates involves epigenetic reprogramming through histone post-translational modifications, particularly by histone methyltransferases, that enhance the expression of antimicrobial genes during re-infection (Netea et al., 2016, Pereira et al., 2016). Interestingly, most studies in this field have been performed in vitro with differentiated immune cells such as monocytes, macrophages or natural killer cells (Netea et al., 2016, Pereira et al., 2016). However, these cell types are already trained for immune function. Investigating the details of innate immune memory in undifferentiated cell types, such as stem cell-like neoblasts, is more challenging. Planarians are a classic model system for the study of adult wound healing and tissue regeneration (Reddien, 2013, Elliott and Sanchez Alvarado, 2013). These free-living members of the phylum Platyhelminthes contain a persistent pool of adult pluripotent stem cells, termed neoblasts, that are capable of producing all cell types and regenerating all types of tissues (Wagner et al., 2011). The ablation of neoblasts via irradiation or specific RNA-based depletion of essential gene products compromises the regenerative capacity of these animals (Reddien et al., 2005b).

Planarians represent a remarkable system with an unmatched capacity to fight infectious agents, including *S. aureus*, indicating the presence of remarkably efficient but uncharacterized innate immunity (Abnave et al., 2014). Notably, several new components of the innate immune system that are conserved in humans and absent from Ecdysozoa (e.g., flies and nematodes) were discovered by studying this model organism (Abnave et al., 2014). The varied abilities of planarians underscore the value of studying microbial defences in this model organism to identify components that are common between innate immunity and regeneration processes.

## 2 Material and Methods

### 2.1 Planarians

*Schmidtea mediterranea* (asexual clonal line ClW4) were maintained at 20 °C in static culture in autoclaved water as previously described (Cebria and Newmark, 2005). The water was changed every two days, and the planarians were maintained without antibiotics. The animals were fed once per week with homogenized calf liver and were starved for at least 1 week prior to experiments.

### 2.2 Bacterial Strains

*Staphylococcus aureus* (ATCC25923) was grown on blood agar plates (BioMerieux SA, Fr). *Legionella pneumophila* (ATCC33152) was grown on buffered charcoal yeast extract (BCYE) agar (Oxoid). *Mycobacterium avium* (BAA-535) was grown on 7H10 agar (Becton Dickinson).

### 2.3 Worm Feeding With Bacterial Pathogens

For all experiments, planarians were infected with 1 × 10^9^ CFU of *S. aureus*, *L. pneumophila*, or *M. avium* as previously described. The planarians were fed with the bacterial pathogens using a protocol adapted from a dsRNA feeding method (Reddien et al., 2005a, Abnave et al., 2014). Briefly, bacterial pellets containing 1 × 10^9^ CFU were suspended in 30 μL of homogenized calf liver, mixed with 15 μL of 2% ultra-low-gelling-temperature agar and 0.7 μL of red food colouring, and allowed to solidify on ice. Room temperature (RT) solidified food was fed to the planarians. After 6 h (defined as day 0, primo-infection) of feeding, the planarians were washed extensively and used for experiments. Thirty days later, the planarians that underwent primo-infection were re-infected with the same bacterial species at the same concentrations used for the primo-infection and then processed as described above. For some experiments, planarians were treated 24 h prior to infection with 1 mM 5′-deoxy-5′-(methylthio) adenosine (MTA, Sigma). The MTA was maintained throughout the duration of the experiments.

### 2.4 CFU Counting

Planarians were collected and homogenized in 20 μL of phosphate-buffered saline. The lysate was passed 5 times through a sterile syringe with a 29 G needle to disrupt planarian tissue clumps, and CFUs were counted after plating 10-μL serial dilutions onto agar plates containing appropriate bacterial growth media.

### 2.5 Gene Prediction and Phylogenetic Tree Construction

*Smed-PGRP-2*, a homologue of *hs-PGLYRP-2*, was identified using the SmedGD genome database (http://smedgd.neuro.utah.edu/, parameters e-value 1e-5) (Robb et al., 2015) with the sequence Smed Unigene SMU15000352 (e-value 8e-53). A phylogenetic tree for PGRP-2 was constructed as follows. From the inferred Smed-PGRP-2 sequence, a dataset of putative homologous sequences was built a BLAST (Altschul et al., 1997) search of the NCBI non-redundant (NR) database. Raw data were manually filtered to eliminate potentially non-homologous sequences that would disturb alignments. Alignments were generated using MUSCLE and refined manually (Edgar, 2004). We used the maximum likelihood method in PhyML to perform phylogenetic reconstruction (Guindon and Gascuel, 2003). Branch statistical supports were estimated using the Shimodaira-Hasegawa-like test (SH) (Anisimova and Gascuel, 2006).

### 2.6 Cloning

To generate an RNAi library, cDNA from *S. mediterranea* was amplified via PCR using primers designed with Primer3 (http://primer3.sourceforge.net/). The primers contained an attB recombination sequence (CATTACCATCCCG). The obtained PCR products were cloned into *E. coli* strain HT115 as described elsewhere (Timmons et al., 2001, Newmark et al., 2003, Reddien et al., 2005b). Targeted transcript sequences were extracted between the 3′ end of the 5′ primer and the 5′ end of the 3′ primer and used for cloning. The extracted sequences were then cut into 21-mers using a sliding window of 1 nucleotide, and all possible RNAi sequences were generated. Each putative RNAi was aligned to the planarian transcriptome using BLAST (Altschul et al., 1990) with a word size of 21; only perfect matches were considered. For each transcript for which an RNAi was designed, the theoretical target accuracy was calculated based on the number of RNAi sequences that matched the target divided by the total number of generated RNAi sequences. The number of theoretical off-target events was equal to 0, thus giving a target accuracy of 100%, which strongly suggests a gene-specific effect but does not exclude the possibility of an off-target effect.

### 2.7 Delivery of dsRNA and RT-qPCR

dsRNAs were delivered as previously described (Newmark et al., 2003, Reddien et al., 2005a). Briefly, worms were fed three times over the course of six days with a planarian artificial food mixture containing homogenized liver, dsRNA-expressing *E. coli* cells, ultra-low gelling temperature agarose, and food colouring. The quality of gene silencing in naive worms was validated by performing real-time RT-qPCR three days after the last RNAi treatment. When gene silencing occurred before infection, naive worms were silenced as described above and then infected with bacteria three days after the last RNAi treatment. When gene silencing occurred after primo-infection, instructed worms were silenced fifteen days after primo infections as described above and then re-infected with bacteria nine days after the last RNAi treatment. Gene expression in animals was measured by performing real-time RT-qPCR as described previously by (Abnave et al., 2014, Forsthoefel et al., 2012). Briefly, total RNA samples (one animal per sample) were prepared using TRIzol according to the manufacturer's instructions (Invitrogen). The following primers were used for RT-qPCR: *Smed-Morn2* (CGTCAAGGGAAAGGTATTAGCG, GTCGCCTTCATATTTTGCACCA), *Smed-p38 MAPK* (GCGAGGCAGACAGATGAAGA, GCGTGTAAACAATTCGGCCA), *Smed-setd8-1* (CAAGCAAGATCCCAGCAAAGG, GGTTCAGTAGACGCCCCAAT), *Smed-PGRP-2* (CACGGAAAGAATGGGGAGCT, TGCTTTGGTTCATAATGAGGCC), and *Smed-PGRP-1, -3, -4* from (Arnold et al., 2016). Then, gene expression was calculated as follows. Mean Ct values were normalized to the control housekeeping gene *Smed-Ef2* (CAGCCAGTAGCTTTAAGCGATG, ACTCTCAACGCTGCTGTCACTTC) (Solana et al., 2012, Forsthoefel et al., 2012, Solana et al., 2013, Fernandez-Taboada et al., 2010) and were used to calculate relative expression (Forsthoefel et al., 2012). With the exception of experiments investigating at expression kinetics, gene expression was assessed 12 hours post-infection for *Smed-p38 MAP-Kinase*, 6 hours post-infection for *Smed-morn2,* and 24 hours post-infection with bacteria for *Smed-setd8-1* and *Smed-PGRP-2, -1, -3,* and *-4*.

### 2.8 In Situ Hybridization and Probe Synthesis

Whole-mount in situ hybridization was performed as previously described (Pearson et al., 2009, King and Newmark, 2013). All animals were 1 to 2 mm in length and were size-matched between the experimental and control groups for *S. mediterranea* infection. The animals were imaged using a Leica M165FC stereomicroscope (Leica, Heidelberg). Images were processed using Adobe Photoshop CS5 software, and figures were assembled using Adobe Illustrator Artwork 15.0.

### 2.9 Planarian Irradiation

Planarians were irradiated at 60 Gy (4 Gy/min) to remove neoblasts using a Synergy S MLC80 Elekta Linac device (Radiotherapy Service, Timone Hospital, Marseille, France) as previously described (Wagner et al., 2011). The quality and efficiency of irradiation were validated by detecting the absence of *Smedwi-1* expression via in situ hybridization five days after irradiation. For some experiments, gene expression in irradiated naive worms was determined by performing RT-qPCR five days after irradiation. For some experiments, irradiated worms were infected five days after infection, and then gene expression was assessed by performing RT-qPCR at 24 hours post-infection for *Smed-p38 MAP-Kinase*, *Smed-morn2*, *Smed-setd8-1* and *Smed-PGRP-2.*

### 2.10 Flow Cytometry

Dissociated *S. mediterranea* cells were prepared as previously described (Hayashi et al., 2006). A stem cell population gate was defined using cells isolated from planarians irradiated at 60 Gy (4 Gy/min) and cells from planarians that had not been exposed to irradiation. Cells were stained with Hoechst red and Hoechst blue according to previously described methods (Hayashi et al., 2006). Lysine methylation levels in stem cell populations from animals that underwent silencing for genes of interest prior to challenge with bacteria for 36 h were then analysed after labelling the cells with antibodies against lysine methylation (Abcam) or an isotype control (Abcam). Secondary antibodies were conjugated to Alexa Fluor-647 (Invitrogen). Flow cytometry acquisition and analysis were performed using a Canto II flow cytometer from BD Biosciences and FACSDiva Software. Images were post-processed in Adobe Photoshop, and figures were assembled in Adobe Illustrator Artwork 15.0.

### 2.11 Fluorescent-activated Cell Sorting (FACS)

Cells suspensions were prepared for FACS as previously described (Hayashi et al., 2006). Briefly, worms (sixty animals per experimental condition, n = 2) were challenged with *S. aureus* for different periods of time and then collected and dissociated. Cell suspensions were characterized by performing selective staining with fluorescent dyes. Nuclear DNA was stained using Hoechst 33,342, calcein AM was applied to determine the cellular volume of viable cells, and propidium iodide was used to stain and eliminate dead cells. Cell suspensions were incubated in 5/8 Holtfreter's solution and pelleted by centrifugation. The cells were then resuspended in 5/8 Holtfreter's solution. Neoblasts were sorted using a BD FACS Jazz instrument from BD Biosciences. After collection, the neoblasts were stored at − 20 °C in TRIzol according to the manufacturer's instructions (Invitrogen) for subsequent RNA extraction.

### 2.12 Planarian Tissue Transplantation

Tissue transplantation was performed as previously described (Guedelhoefer and Sanchez Alvarado, 2012) (see also Fig. S2E). Briefly, animals (2 cm in size) were anaesthetized using chilled chloretone solution (0.2%). The worms were oriented ventral side down, and then a piece of tissue was withdrawn using a glass capillary tube (0.75 mm inner diameter) from a donor (*S. aureus* instructed animals or naive animals) that was irradiated (60 Gy; 4 Gy/min) or not irradiated. The tissue (instructed tissue or naive tissue) was then transplanted (5 days after irradiation) onto a host (naive animal) in a hole made by removing a piece of tissue using a glass capillary tube (0.7 mm inner diameter). The transplanted animals were covered with a piece of rolling paper wetted with casein-saturated Holtfreter's solution and then encased and covered with filter paper wetted with casein-saturated Holtfreter's solution. The transplanted animals were maintained overnight at 10 °C and then transferred to a 20 °C incubator for experiments. Animals were infected with *S. aureus* fifteen days after transplantation.

### 2.13 In silico Analysis

RNA sequencing raw data from planarian tissue and FACS-purified neoblasts from Labbe et al. (Labbe et al., 2012) were downloaded from the GEO database under the accession number GSE37910. The FastA file was converted into a nucleotide database through a BLAST search. A selection of gene sequences (*Smed-PGRP-2, Smed-setd8-1, Smed-morn2,* and *Smed-p38 MAP-kinase*) was searched against this newly formed database using BLASTN. The best BLAST hits were defined as having an identity higher than 30% and a coverage of at least 70%. Expression values were retrieved from the GEO database under the accession number GSE37910. The tabulated format was parsed using an in-house PERL script to obtain expression values (cRPKM) for the best BLAST hits obtained from the selected sequences.

### 2.14 Statistical Analysis

Results are expressed as the mean ± SD and were analysed using the nonparametric Mann-Whitney *U* test. Differences were considered significant at p < 0.05.

## 3 Results

### 3.1 *Smed-PGRP-2* Expression Drives Heightened Resistance in Re-infection by *Staphylococcus aureus*

**Fig. 1:**
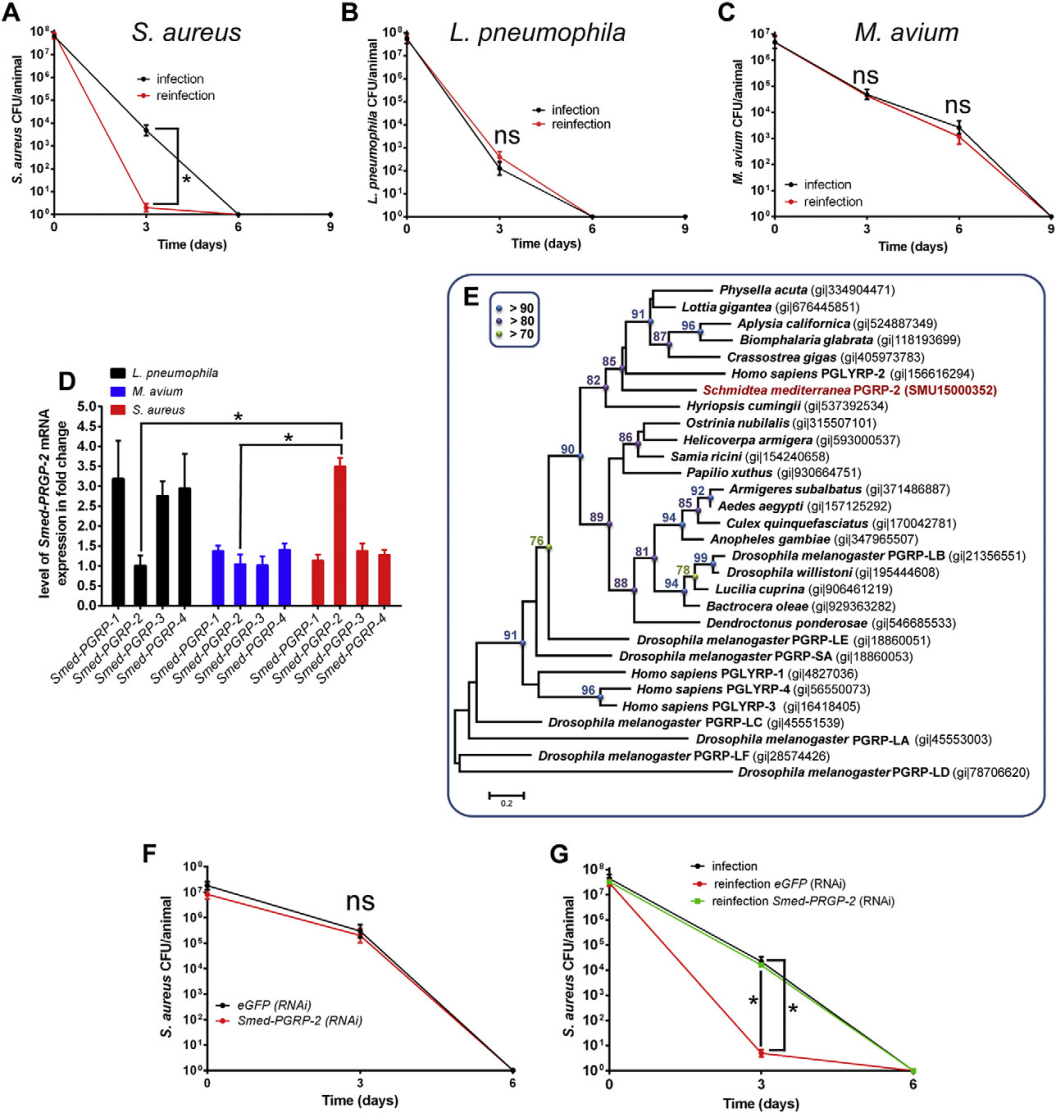
*Smed-PGRP-2* expression drives heightened resistance in re-infection by *Staphylococcus aureus.* (A, B, and C) *S. mediterranea* were infected (primo-infection) with (A) *S. aureus*, (B) *L. pneumophila*, or (C) *M. avium* and then re-infected thirty days after primo-infection with the same bacteria used for primo-infection. Instructed immunity was induced by infection with (A) *S. aureus* but not with (B) *L. pneumophila* or (C) *M. avium*. The results are expressed as the mean ± SD (ten animals per time point, n = 3, *p < 0.05). (D) *Smed-PGRPs* expression levels in planarians challenged with *L*. *pneumophila*, *S. aureus*, or *M. avium* were evaluated 24 hours post-bacterial challenge by performing RT-qPCR (10 animals per experimental condition, n = 3). (E) Phylogenetic reconstruction of *Smed-PGRP-2*. Symbols indicate SH support above > 90 (blue circle), > 80 (purple circle), and > 70 (green circle). The scale bars represent the average number of substitutions per site. (F) *S. mediterranea* was subjected to RNAi-mediated silencing of *Smed-PGRP-2*; then, animals were infected with *S. aureus* three days after the last RNAi treatment, and bacterial behaviour was monitored by performing CFU counting. Silencing of *Smed-PGRP-2* did not affect the ability of planarians to eliminate *S. aureus* during primo-infection. The results are expressed as the mean ± SD (five animals per time point, n = 3). The efficiency of *Smed-PGRP-2* knockdown was confirmed (Fig. S1C). (G) Fifteen days after primo-infection, worms were treated with *Smed-PGRP-2 (RNAi)* prior to re-infection with *S. aureus* thirty days after primo-infection. *Smed-PGRP-2* knockdown abrogated the instructed immunity. The results are expressed as the mean ± SD (ten animals per time point, n = 3, *p < 0.05). The efficiency of *Smed-PGRP-2* knockdown was confirmed (Fig. S1D). All results were analysed using the nonparametric Mann-Whitney *U* test. Differences were considered significant at p < 0.05.

We set out to investigate the molecular basis of planarian immunity to *Staphylococcus* re-infection. We assessed the ability of animals to produce a state of resistance to *S. aureus* after primo-infection, hereafter referred to as instructed immunity. *Schmidtea mediterranea (Smed)* naïve worms that were infected by *Staphylococcus aureus* cleared the infection in < 15 days (Fig. 1A, black lane)*.* Thirty days after primo-infection, the worms were re-infected. The animals displayed a higher rate of pathogen clearance, with resolution of the infection already apparent at 3 days (2 ± 1 CFU/infected animal compared to 5 × 10^3^ ± 3 × 10^2^ CFU/naïve animal) (Fig. 1A, red lane). We concluded that planarians display a form of instructed immunity that confers higher resistance to re-infection. This form of instructed immunity was transient for a period ranging from 15 to 45 days after primo-infection (Fig. S1A). We next assessed planarian instructed immunity against *Legionella pneumophila* or *Mycobacterium avium*. Planarians infected by *Legionella pneumophila* (Fig. 1B, black lane) and *M. avium* (Fig. 1C, black lane) cleared the infection in 6 to 9 days post-challenge, respectively. In contrast to infection by *S. aureus*, we did not observe enhanced clearance of *L*. *pneumophila* or *M. avium* during re-infection (Fig. 1B and C, red lanes). These results suggest a specificity of instructed immunity involving elements of innate immunity. This form of immunity largely relies on the recognition of pathogen-associated molecular patterns (PAMPs) by host innate immune receptors, which leads to the induction of conserved anti-microbial signalling pathways (Ferrandon et al., 2007). Despite the critical role of innate immunity, little is known about the molecular components at work during bacterial re-infection. Peptidoglycan recognition proteins (PGRPs), known as PGLYRPs in vertebrates (Dziarski and Gupta, 2006), are critical receptors in the recognition of gram-positive bacteria (Fournier and Philpott, 2005). Planarians express four PGRP-like receptors, referred to as *Smed-PGRP-1, -2, -3,* and *-4* (Arnold et al., 2016). We analysed by RT-qPCR the expression of all four receptors after challenge of planarians with *S. aureus* (Fig. 1D and S1B)*. S. aureus* specifically induced the expression of *Smed-PGRP-2* (Fig. 1D). *Smed-PGRP-2 mRNA* showed maximal expression 24 hours post-challenge (Fig. S1B). Interestingly, in the evolutionary tree, Smed-PGRP-2 is located close to *H. sapiens* PGRPLY-2, with 46% identity at the amino acid level (Fig. 1E). We then analysed the expression of *Smed-PGRPs* in worms infected 24 hours by *L*. *pneumophila* and *M. avium.* No expression of the four *Smed-PGRP* receptors was observed in planarians infected by *M. avium* (Fig. 1D). Worms infected by *L*. *pneumophila* expressed *Smed-PGRP-1, -3,* and *-4* only (Fig. 1D). We thus hypothesized a critical involvement of Smed-PGRP-2 in establishing the specific immune resistance to *S. aureus* re-infection. Treatment of worms with *Smed-PGRP-2 (RNAi)* prior to infection had no impact on the efficiency of clearance of *S. aureus* (Fig. 1F). The silencing of *Smed-PGRP-2* was verified (Fig. S1C). By contrast, animals that were treated with *Smed-PGRP-2* (*RNAi)* 15 days after primo-infection prior to re-infection 15 days later displayed no enhanced capacity to clear *S. aureus* (Fig. 1G and S1D). Indeed, the clearance of bacteria during the re-infection phase was similar to that recorded during primo-infection. Together, these data reveal a critical role of Smed-PGRP-2 expression in *S. aureus*-infected animals for establishing a state of heightened resistance to *S. aureus* infection.

### 3.2 Neoblast-based Heightened-resistance to *S. aureu*s Re-infection

**Fig. 2:**
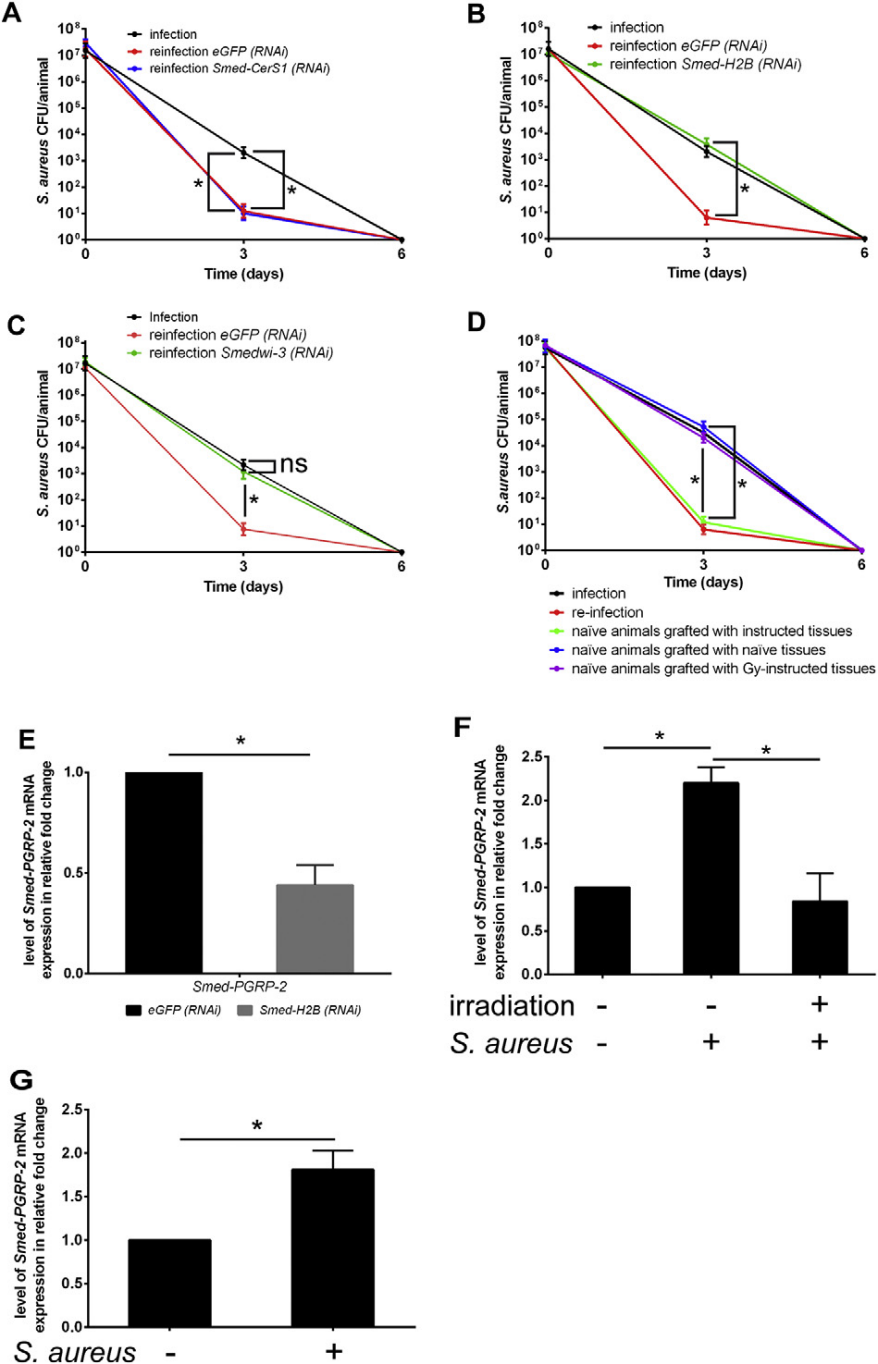
Neoblast-based heightened resistance to *S. aureu*s re-infection. (A) Fifteen days after primo-infection, worms were treated with *Smed-CerS1 (RNAi)* prior to re-infection with *S. aureus* thirty days after primo-infection. Phagocyte depletion via *Smed-CerS1 (RNAi)* did not interfere with the establishment of instructed immunity. The results are expressed as the mean ± SD (five animals per time point, n = 3, *p < 0.05). *Smed-CerS1* depletion was verified by performing RT-qPCR (Fig. S2A). (B) Fifteen days after primo-infection, worms were treated with *Smed-H2B* RNAi prior to re-infection with *S. aureus* thirty days after primo-infection. Neoblast depletion via *Smed-H2B (RNAi)* (green lane) in instructed *S. mediterranea* abrogated the animals' immunity compared to those exposed to control *eGFP (RNAi)* (red lane)*.* The results are expressed as the mean ± SD (ten animals per time point, n = 3, *p < 0.05). *Smed-H2B* depletion was verified by performing ISH and RT-qPCR (Fig. S2B and C). (C) Fifteen days after primo-infection, worms were treated with *Smedwi-3 (RNAi)* prior to re-infection with *S. aureus* thirty days after primo-infection. Stem cell depletion via *Smedwi-3 (RNAi)* in instructed *S. mediterranea* abrogated the animals' immunity compared to those exposed to control *eGFP (RNAi)* (red lane)*.* The results are expressed as the mean ± SD (five animals per time point, n = 2, *p < 0.05). *Smedwi-3* depletion was verified by performing RT-qPCR (Fig. S2D). (D) Planarians were infected and then ten days later irradiated (60 Gy) or not. Five days post-irradiation a ragments of irradiated instructed tissue were grafted onto naïve worms. Then, chimeric animals were re-infected with *S. aureus* fifteen days later (see also Fig. S2E). Improved bacterial clearance was observed after grafting (green lane). Irradiation at 60 Gy abolished the instructed immunity (purple lane). The results are expressed as the mean ± SD (ten animals per time point, n = 3, *p < 0.05). The quality of the irradiation was confirmed by detecting *Smedwi-1* mRNA via ISH (Fig. S2F). (E) Planarians subjected to RNAi-mediated silencing of *Smed-H2B* were challenged with *S. aureus* three days after the last RNAi treatment. *Smed-PGRP-2* expression was evaluated by performing RT-qPCR 24 h after *S. aureus* challenge. *Smed-PGRP-2* mRNA expression was decreased by *Smed-H2B* knockdown. The results are expressed as the mean ± SD (five animals per experimental condition, n = 3, *p < 0.05). (F) Planarians underwent neoblast depletion via irradiation at 60 Gy and were then challenged five days later with *S. aureus* for 24 h. *Smed-PGRP-2* expression levels were determined by performing RT-qPCR. Neoblast depletion inhibited *Smed-PGRP-2* expression in response to *S. aureus* challenge. The results are expressed as the mean ± SD (five animals per experimental condition, n = 3, *p < 0.05). (G) Planarians were infected with *S. aureus* for 24 h, and then neoblasts were sorted via FACS. *Smed-PGRP-2* expression in the sorted neoblasts was analysed by performing RT-qPCR. *S. aureus* induced *Smed-PGRP-2* expression in neoblasts sorted from planarians*.* The results are expressed as the mean ± SD (n = 3, *p < 0.05). All results were analysed using the nonparametric Mann-Whitney *U* test. Differences were considered significant at p < 0.05.

We next focused on defining the contributing roles of neoblastic and phagocytic cells to instructed immunity. *Smed-CerS1* is a gene involved in the maintenance of phagocytic cells (Forsthoefel et al., 2012)*.* We thus analysed the role of phagocytic cells by selective depletion of this cell type via *Smed-CerS1 (RNAi)* treatment 15 days after primo-infection. Animals were then challenged with *S. aureus* 30 days after primo-infection and pathogen clearance was determined by CFU counting (Fig. 2A). The silencing of *Smed-CerS1* was verified (Fig. S2A). Treatment of re-infected animals with *Smed-CerS1 (RNAi)* had no impact on the efficiency of bacterial clearance (Fig. 2A). We thus turned toward the role of neoblasts (Fig. 2B and C). *Smed*-*H2B* is an essential gene that encodes a neoblast-specific histone isoform required for the maintenance of this cell type. Silencing of *Smed-H2B* has been reported to lead to depletion of neoblasts and associated defects in tissue regeneration after wounding (Solana et al., 2012, Guo et al., 2006, Reddien, 2013). We treated animals with *Smed*-*H2B* (*RNAi)* 15 days after primo-infection and verified the efficiency of the silencing by in situ hybridization (ISH) (Fig. S2B) and by RT-qPCR (Fig. S2C). We next challenged *Smed*-*H2B* depleted animals with *S. aureus* 30 days after primo-infection and monitored pathogen clearance. Remarkably, under these experimental conditions, the kinetics of bacterial clearance from the re-infected animals was indistinguishable from that during primo-infection (Fig. 2B, green lane). Indeed, *Smed*-*H2B* (*RNAi)*-treated animals no longer exhibited the heightened bacterial clearance characteristic of instructed immunity. As a second approach, we depleted neoblasts by silencing either *Smedwi-2* or *Smedwi-3* (Palakodeti et al., 2008) following the same experimental procedure. Silencing of *Smedwi-2* triggered early death in 100% of re-infected worms by 5 days after infection. This induction of death may be linked to the fact that *Smedwi-2* is also expressed in neuronal tissues, including the brain (Reddien et al., 2005b). Therefore, this approach was inconclusive. By contrast, when we silenced *Smedwi-3* after primo-infection, re-infected animals no longer displayed heightened clearance of *S. aureus* (Fig. 2C and S2D), and the kinetics of pathogen clearance was similar to that measured during primo-infection. These experiments are the first to indicate a critical role of neoblasts in the establishment of enhanced resistance to *S. aureus*.

Planarians are a remarkable model system that offers the possibility to perform tissue grafting to assess the role of neoblasts in tissue regeneration (Guedelhoefer and Sanchez Alvarado, 2012). We therefore examined whether instructed immunity can be transferred into naïve animals via tissue grafting using the classic procedure outlined in Fig. S2E as previously established by (Guedelhoefer and Sanchez Alvarado, 2012). A piece of calibrated tissue was withdrawn from an animal donor, i.e., a naïve animal or animal primo-infected by *S. aureus*. The tissue was grafted onto a naïve planarian. The animals were allowed to recover for 15 days prior to infection. At the time of infection, the animals had received cells from naïve donors or donors infected 30 days ago. Remarkably, the grafted animals displayed an enhanced capacity to clear bacteria, with values of 12 ± 8 CFU/naïve animals grafted with instructed tissue (green lane) versus 5 × 10^4^ ± 3 × 10^3^ CFU/naïve animals grafted with naïve tissue (blue lane) (Fig. 2D). As a control, we irradiated instructed worms to eliminate neoblasts prior to transplant (Wagner et al., 2011). Neoblast elimination was monitored by ISH using the *smedwi-1* gene, which encodes a PIWI-like protein, as a probe (Reddien, 2013, Solana et al., 2012) (Fig. S2F)*.* Grafting of irradiated tissue from infected animal donors failed to promote instructed immunity in naive worms (Fig. 2D, purple lane). Collectively, these data indicate that the information from instructed immunity can be transferred by transplantation of tissue from infected animals, further supporting a critical role of neoblasts. We then delineated the role of neoblasts in the expression of *Smed-PGRP-2* in primo-infected worms. Naive planarians were treated with *Smed-H2B (RNAi)* to deplete the neoblast population, followed by *S. aureus* challenge. In these animals, we recorded a 55% decrease in *Smed-PGRP-2* expression (Fig. 2E). Depletion of neoblasts by irradiation prior to infection by *S. aureus* ablated the *Smed-PGRP-2* response (Fig. 2F), and the level of *Smed-PGRP-2* was similar to that measured in control animals*.* Similarly, ISH revealed an absence of *Smed-PGRP-2* mRNA expression in tissues of infected animals irradiated 5 days before infection (Fig. S2G). mRNA sequencing data reported by (Labbe et al., 2012) (GSE37910) indicates that *Smed-PGRP-2* is expressed in sorted neoblasts (Table S1). This prompted us to assess *Smed-PGRP-2* mRNA expression in neoblasts from naive and *S. aureus*-infected worms using fluorescent-activated cell sorting (FACS) (Hayashi et al., 2006) (Fig. S2H). Comparison of mRNA expression levels in sorted neoblasts from naïve and infected worms revealed a 2-fold increase in *Smed-PGRP-2* gene expression in neoblasts 24 h after primo-infection (Fig. 2G). Taken together, our data establish a critical role of neoblasts in *S. aureus*-mediated induction of *Smed-PGRP-2* expression to confer enhanced protection during re-infection.

### 3.3 *Smed-setd8-1* Expression Controls Heightened Resistance to *S. aureus* Re-infection

**Fig. 3:**
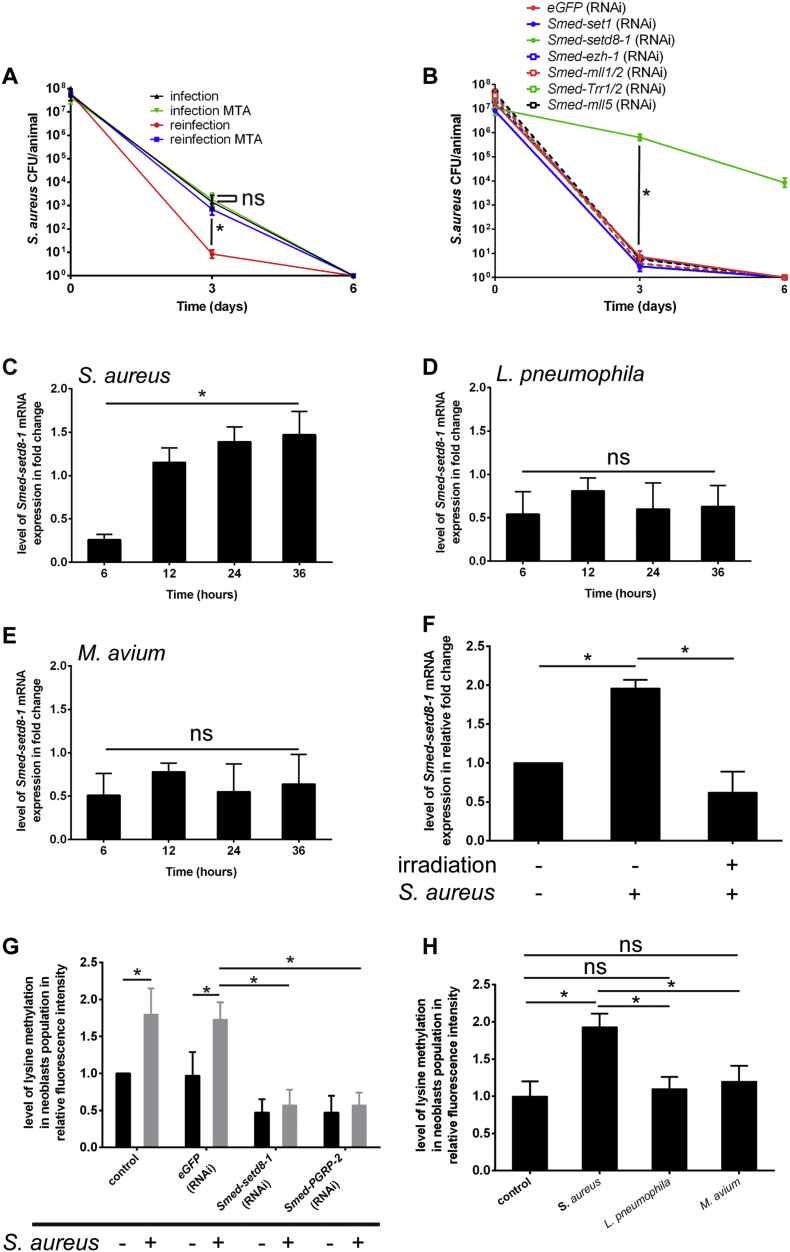
*Smed-setd8-1* expression controls heightened resistance to *S. aureus* re-infection. (A) Planarians were treated with MTA (1 mM) or left untreated, infected (primo-infection) with *S. aureus* (green lane and black lane), and then re-infected 30 days later with *S. aureus* (blue lane and red lane). MTA treatment abrogated the establishment of instructed immunity. The results are expressed as the mean ± SD (ten animals per time point, n = 2, *p < 0.05). (B) Fifteen days after primo-infection, worms underwent RNAi knockdown for histone methyltransferases prior to re-infection with *S. aureus* thirty days after primo-infection. *Smed-setd8-1* knockdown inhibited the establishment of instructed immunity. The results are expressed as the mean ± SD (ten animals per time point, n = 3, *p < 0.05). Knockdown efficiencies were confirmed (Fig. S3A). (C, D, E) *Smed-setd8-1* expression was measured by performing RT-qPCR in worms challenged with (C) *S. aureus*, (D) *L. pneumophila,* or (E) *M. avium*. *Smed-setd8-1* expression was induced by (C) *S. aureus* but not by (D) *L. pneumophila* or (E) *M. avium.* For C, the results are expressed as the mean ± SD (five animals per time point, n = 3, *p < 0.05). For D and E, the results are expressed as the mean ± SD (five animals per time point, n = 2), and the differences are not significant. (F) Planarians were irradiated at 60 Gy, and then the animals were challenged five days later with *S. aureus* for 24 h. Neoblast depletion inhibited *Smed-setd8-1* expression, as determined by RT-qPCR, in response to *S. aureus* challenge. The results are expressed as the mean ± SD (five animals per experimental condition, n = 3, *p < 0.05). (G) Lysine methylation levels in neoblast populations from animals silenced for *Smed-sted8.1* or *Smed-PGRP-2* and then challenged (three days after the last RNAi treatment) by *S. aureus* were evaluated by performing flow cytometry 36 h after bacterial challenge (see also Fig. S3E and F). *S. aureus* induced lysine methylation, which was abrogated by *Smed-setd8-1* knockdown and *Smed-PGRP-2* knockdown. The results are expressed as the mean ± SD (twenty animals per experimental condition, n = 3, *p < 0.05). (H) Lysine methylation levels in neoblast populations from animals infected for 36 h with *L*. *pneumophila* or *M. avium* were evaluated by flow cytometry. Neither *L*. *pneumophila* nor *M. avium* induced a significant increase in lysine methylation in neoblasts. The results are expressed as the mean ± SD (twenty animals per experimental condition, n = 3, *p < 0.05). All results were analysed using the nonparametric Mann-Whitney *U* test. Differences were considered significant at p < 0.05.

Trained immunity in vertebrates involves histone methylation. This prompted us to explore whether components of the epigenetic machinery, such as histone methyltransferases, might contribute to instructed immunity. Several studies have identified different types of methyltransferases in the planarian genome that are involved in epigenetic reprogramming via post-translational modification of histones (Hubert et al., 2013, Wagner et al., 2012, Robb and Sanchez Alvarado, 2012). We first treated worms with 5′-deoxy-5′-(methylthio) adenosine (MTA), a broad inhibitor of histone methyltransferases (Quintin et al., 2012). MTA was administered 24 h before the experiment and continued during the infection phase. MTA did not affect *S. aureus* clearance during primo-infection (Fig. 3A). By contrast, the kinetics of bacterial clearance in re-infected worms was similar to that in primo-infected worms (Fig. 3A). These initial experiments indicated a role of histone methyltransferases in the establishment of instructed immunity. Therefore, we conducted a candidate approach to evaluate the role of each histone methyltransferase in the heightened resistance of planarians to *S. aureus* (Fig. 3B). RNAi-mediated depletions were carried out 15 days after primo-infection and quantified by RT-qPCR (Fig. S3A). When these animals were re-infected, no effect of depletion was observed except for *Smed-setd8-1*, the depletion of which dramatically reduced the capacity of worms to clear *S. aureus* (Fig. 3B). By contrast and in good agreement with a specific role of *Smed-setd8-1* in instructed immunity, *Smed-setd8-1* depletion before primo-infection had no impact on bacterial clearance efficiency (Fig. S3B). As a complementary approach, we recorded the kinetics of *Smed-setd8-1* mRNA expression in animals infected with *S. aureus, L. pneumophila* or *M. avium* (Fig. 3C–E and S3C). During primo-infection, *S. aureus* induces the expression of *Smed-setd8-1* at the difference of *L*. *pneumophila* and *M. avium*. Interestingly, analysis of publicly available mRNA sequencing data (GSE37910) reported by (Labbe et al., 2012) showed that *Smed-setd8-1* is expressed in neoblasts (Table S1). We depleted neoblasts from naive animals by irradiation and measured *Smed-setd8-1* expression five days after irradiation. We recorded a 60% reduction of the expression of *Smed-setd8-1* mRNA (Fig. S3D). When the analysis was conducted in infected worms, we observed an absence of expression of *Smed-setd8-1* mRNA (Fig. 3F). These data functionally implicate *Smed-setd8-1* histone methyltransferase in neoblast-mediated instructed immunity. To provide a link between the expression of Smed-setd8-1 in neoblasts and its lysine methyltransferase activity, we measured the content of methylated lysine in this cell population by flow cytometry 36 h after infection using a method defined by (Hayashi et al., 2006) (Fig. S3E). We recorded a 1.8-fold increase in methylated lysine content in neoblasts of primo-infected animals compared to naïve animals (Fig. 3G and S3F). This increase was abrogated by knockdown of *Smed-setd8-1* (Fig. 3G and Fig. S3F) or *Smed-PGRP-2* (Fig. 3G) 36 h before infection. By contrast, increased lysine methylation in neoblasts was not observed in animals infected with *L*. *pneumophila* or *M. avium* (Fig. 3H). Collectively, our results establish the critical involvement of *Smed-setd8-1* in enhanced clearance of *S. aureus* during the re-infection phase linked to increased lysine methylation in neoblasts.

### 3.4 Smed-PGRP-2 Promotes *Smed-setd8-1*-dependent Induction of Anti-microbial Gene Responses

**Fig. 4:**
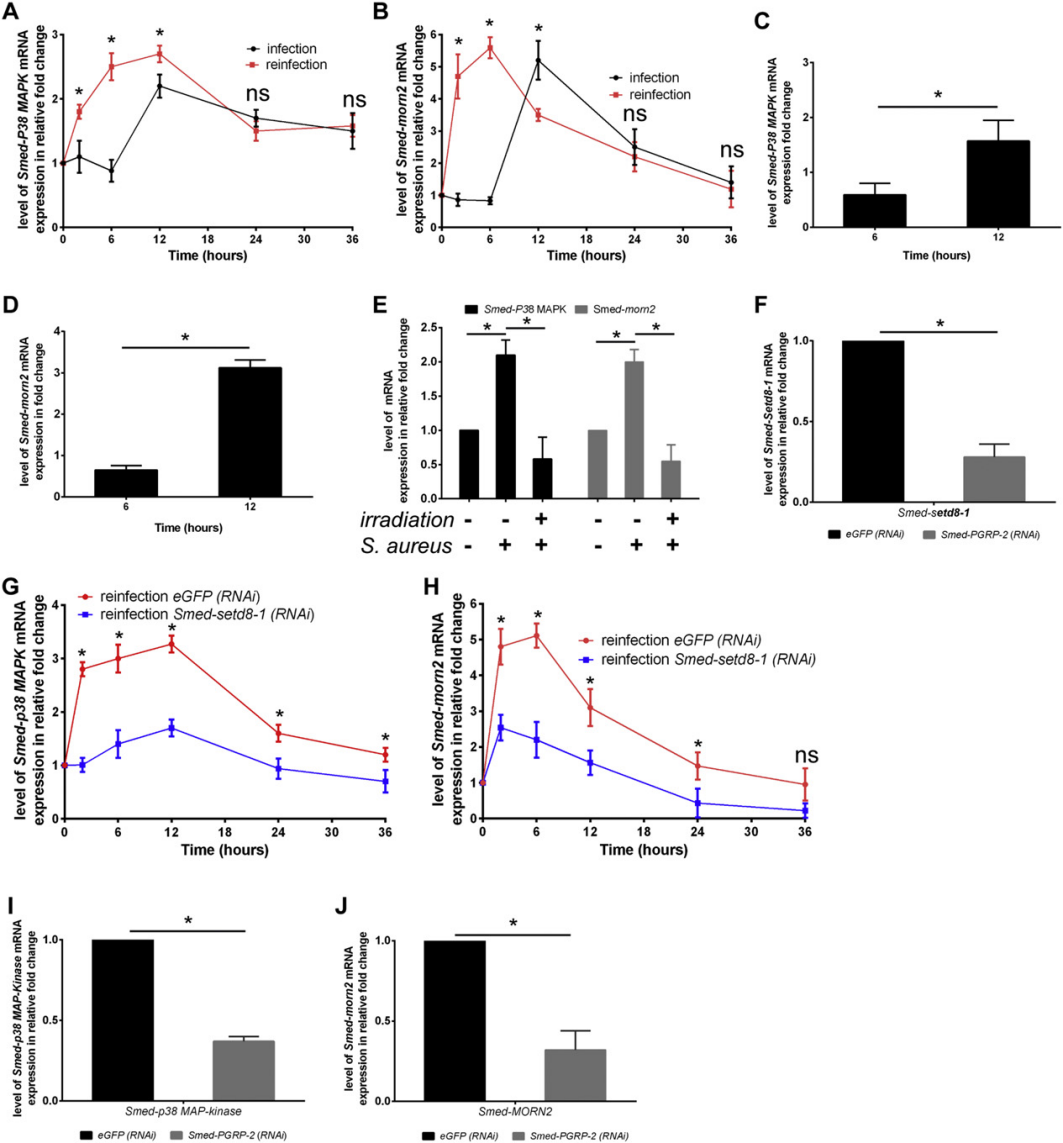
Smed-PGRP-2 promotes *Smed-setd8-1*-dependent induction of anti-microbial gene responses. (A and B) *Smed-p38* MAP kinase (A) and *Smed-morn2* (B) mRNA levels were determined by performing RT-qPCR in animals that were infected and then re-infected thirty days later with *S. aureus.* (A) *Smed-p38* MAP kinase and (B) *Smed-morn2* mRNA were rapidly expressed and showed increased expression during infection recall. The results are expressed as the mean ± SD (five animals per time point, n = 3). (C and D) Planarians were challenged with *S. aureus*, and neoblasts were sorted via FACS. (C) *Smed-p38* MAP kinase and (D) *Smed-morn2* expression in sorted neoblasts were analysed by performing RT-qPCR. Neoblasts isolated from infected animals expressed (C) *Smed-p38* MAP kinase and (D) *Smed-morn2*. The results are expressed as the mean ± SD (n = 3, *p < 0.05). (E) Planarians were irradiated at 60 Gy and then infected five days later with *S. aureus*. *Smed-p38* MAP kinase and *Smed-morn2* expression were determined by performing RT-qPCR 24 h post-challenge. Neoblast depletion inhibited *Smed-p38* MAP kinase and *Smed-morn2* expression in response to *S. aureus*. The results are expressed as the mean ± SD (five animals per experimental condition, n = 3, *p < 0.05). (F) *Smed-setd8-1* mRNA levels in animals silenced for *Smed-PGRP-2* and then challenged with *S. aureus* for 24 h were evaluated by RT-qPCR. *Smed-setd8-1* mRNA expression in *S. aureus*-infected worms was decreased by *Smed-PGRP-2* knockdown. The results are expressed as the mean ± SD (ten animals per experimental condition, n = 3, *p < 0.05). (G and H) Fifteen days after primo-infection, worms were treated with *Smed-setd8-1 (RNAi)* prior to re-infection with *S. aureus* thirty days after primo-infection. (G) *Smed-p38* MAP kinase and (H) *Smed-morn2* mRNA expression were determined by performing RT-qPCR. *Smed-setd8-1* silencing by RNAi significantly diminished the expression of (G) *Smed-p38* MAP kinase and (H) *Smed-morn2* by approximately 50%. The results are expressed as the mean ± SD (ten animals per time point, n = 3, *p < 0.05). (I and J) Animals were silenced for *Smed-PGRP-2*, and then the animals were challenged with *S. aureus* three days after the last RNAi treatment. (I) *Smed-p38 MAP kinase* and (J) *Smed-morn2* mRNA levels were determined by performing RT-qPCR 12 hours and 6 hours post-challenge, respectively. The results are expressed as the mean ± SD (ten animals per experimental condition, n = 3, *p < 0.05). All results were analysed using the nonparametric Mann-Whitney *U* test. Differences were considered significant at p < 0.05.

To determine if instructed immunity against *S. aureus* has an impact on the expression of antimicrobial gene responses, we recorded the expression of p38 MAP kinase mRNA (*Smed-p38 MAPK*), which is induced by bacterial PAMPs in planarians (Pang et al., 2016), and *Smed-morn2*, which controls microtubule-associated protein 1A/1B–light chain 3 (LC3)-associated phagocytic destruction of bacteria (Abnave et al., 2014). *Smed-p38 MAPK* and *Smed-morn2* expression were induced much earlier following re-infection with *S. aureus* than during primo-infection (Fig. 4A and B). By contrast, *L. pneumophila* did not prime the expression of these innate immune executor genes during re-infection (Fig. S4A and 4B). Analysis of publicly available mRNA sequencing data (Labbe et al., 2012) (GSE37910) showed that neoblasts express *Smed-morn2* and *Smed-p38 MAPK* (Table S1)*.* In agreement with these data, we measured an induction of *Smed-p38 MAPK* and *Smed-morn2* mRNA expression in FACS-sorted neoblasts of animals infected with *S. aureus* (Fig. 4C and D). These results show that innate immune executor genes are also expressed in neoblasts, in which primo-infection engages a Smed-setd8-1-dependent signalling cascade of resistance against *S. aureus*. In addition, mRNA expression of these innate immune executor genes was absent in animals that were irradiated 5 days prior to challenge with *S. aureus* for 24 h (Fig. 4E). Our data indicate that primo-infection of planarians by *S. aureus* establishes a transient state of instructed immunity that consists of the implementation of a genetic program of sensitized expression of innate immune executor genes. We next examined the hierarchy of expression of *Smed-PGRP-2* and other components defined in this study. *RNAi*-mediated depletion of *Smed-PGRP-2* prior to animal infection reduced the expression of *Smed-setd8-1* by 72% (Fig. 4F). The RNAi-mediated depletion of *Smed-setd8-1* prior to animal infection had no impact on the expression of *Smed-PGRP-2* during primo-infection (Fig. S4C), while its silencing 15 days after primo-infection decreased the expression *Smed-p38* MAPK (Fig. 4G, blue lane) and *Smed-morn2* (Fig. 4H, blue-red lane) in re-infected animals. Finally, depletion of *Smed-PGRP-2* in infected worms reduced the expression of *Smed-p38 MAPK* by 63% (Fig. 4I) and the expression of *Smed-morn2* by 68% (Fig. 4J) following re-infection. Collectively, our data establish that *Smed-PGRP2* and *Smed-setd8-1* expression are related and facilitate the expression of the innate immune executor genes *Smed-p38* MAPK and *Smed-morn2* during staphylococcal re-infection.

## 4 Discussion

Here, we report that planarians initiate a genetic program of instructed immunity during *S. aureus* infection that allows a sensitized expression of anti-microbial responses upon re-infection to clear the pathogen more efficiently. Mechanistically, we defined the critical role of neoblasts and the expression of the *Smed-PGRP-2* peptidoglycan receptor and the *Smed-setd8-1* histone methyltransferase. These factors promote the expression of the executor genes *Smed-p38 MAPK* and *Smed-morn2*, which display a facilitated expression that is associated with enhanced bacterial clearance. Moreover, we established that *Smed-PGRP-2* controls the induction of *Smed-setd8-1* and the downstream increase in lysine methylation content in neoblasts.

We propose that instructed neoblasts orchestrate the heightened anti-bacterial gene response to *S. aureus*, which comprises *Smed-p38 MAP kinase* and *Smed-morn2*. This raises the question of whether neoblasts also control anti-bacterial responses during primo-infection. In support of this hypothesis, we measured an absence of expression of *Smed-p38 MAPK* and *Smed-morn2* in primo-infected worms that were irradiated. However, irradiated animals retained a capacity to eliminate *S. aureus* similar to that in control worms (not shown). Together, our findings point for neoblasts as central elements in establishing instructed immunity rather than in contributing directly to pathogen clearance. Combined with our functional analysis, these data indicate an unappreciated role of neoblasts in instructing enhanced resistance to *S. aureus* in planarians.

In the current study, we characterized a form of instructed immunity that involves the expression of innate immune genes. We have characterized the critical function of *Smed-PGRP-2* in instructed immunity*.* This peptidoglycan receptor shows close protein sequence homology with human PGRP-2 (PGRPLY-2), which has a more subtle function in immunity than other PGRP family members (Fournier and Philpott, 2005). In line with our findings, PGRP-2 deficiency in human macrophages has no direct impact on inflammation triggered by *S. aureus*, leaving possible a hidden role in recurrent staphylococcal infections (Fournier and Philpott, 2005, Xu et al., 2004). Interestingly, instructed immunity in planarians showed some specificity against *S. aureus* and was linked to *Smed-PGRP-2* expression during primo-infection. Indeed, *S. aureus* did not trigger the expression of *Smed-PGRP-1, -3,* or *-4*. Moreover, we recorded an absence of *Smed-PGRP-2* expression in animals infected with *L*. *pneumophila* and *M. avium*, two bacterial species that do not induce instructed immunity. Interestingly, we defined a hierarchy of innate immune gene expression driven by *Smed-PGRP-2*. Depletion of *Smed-setd8-1* did not affect *Smed-PGRP-2* expression*,* whereas we measured a 72% decrease of *Smed-setd8-1* expression triggered by *Smed-PGRP-2* depletion. We observed that depletion of *Smed-setd8-1* had a dramatic impact on bacterial clearance, which was less efficient than during primo-infection. By contrast, neither marker had an effect on primo-infection, indicating their specific importance in instructed immunity. *Smed*-*setd8-1* depletion did not affect neoblast populations, as indicated by the stable expression of neoblast markers (Onal et al., 2012), but abrogated the expression of *Smed-p38 MAPK* and *Smed-morn2* executor genes, similar to the depletion of *Smed-PGRP-2*. Together, these data indicate that *Smed-setd8-1* has a broader importance in immunity than *Smed-PGRP-2*. A hypothesis is that the heightened anti-bacterial responses controlled by *Smed-setd8-1* signify the need to overcome a desensitization phase following primo-infection in addition to sensitizing the induction of host defences.

Planarians are a particularly well-suited animal model for the study of neoblast function, given the remarkable enrichment of this cell type in these animals (Reddien, 2013). Here, we showed that phagocyte depletion had no impact on instructed immunity. Instead, we found evidence of a critical role of neoblasts in instructed immunity. In support of this idea, we demonstrated that immune memory was abrogated by neoblast depletion via silencing of *Smed-H2B* or *Smedwi-3* or via irradiation. In addition, we demonstrated that immune memory can be grafted onto naïve animals and can be reset by irradiation (Solana et al., 2012, Guo et al., 2006). We also identified an immune function for *Smed-setd8-1*, which is a critical component in the neoblast differentiation that occurs during tissue regeneration (Wagner et al., 2012, Onal et al., 2012). In agreement with this hypothesis, we revealed that both *Smed-setd8-1* and *Smed-PGRP2* expression was induced in sorted neoblasts from infected animals. In addition, we showed that *Smed-setd8-1* expression increased protein methylation in neoblasts in response to infection and that this signalling pathway is controlled by *Smed-PGRP-2.* Based on these data, it appears that overlapping components are involved in immunity and tissue regeneration in planarians. Our findings thus provide a molecular link between the cellular machinery of pathogen perception and the processes that control DNA imprinting. Indeed, Netea et al. previously described the critical role of fungal β-glucan in the induction of epigenetic reprogramming (Netea et al., 2016, Quintin et al., 2012). This reprogramming is accompanied by changes in the histone marks H3K4me1, H3K4me3 and M3K27ac, which in turn induce anti-microbial gene responses (Pereira et al., 2016). In the current study, we extended these findings by showing the critical function of *Smed-setd8-1* and neoblasts in promoting the expression of the anti-bacterial effectors *Smed-p38 MAP kinase* and *Smed-morn2* during re-infection. Interestingly, this protein is homologous in sequence to human SET8, which modulates the H4K20me1 histone code in vertebrates and might thus be implicated in innate immune memory in vertebrates (Nishioka et al., 2002, Talasz et al., 2005, Fang et al., 2002).

Drawing parallels between instructed immunity and trained immunity in vertebrates has been hampered by the lack of information on the mechanisms involved in invertebrate model organisms. Nevertheless, our study in planarians and a study conducted in Drosophila now highlight the importance of PAMPs recognition in invertebrates as described in vertebrate (Pham et al., 2007, Van Der Meer et al., 2015). In both cases, the recognition of PAMPs engages lysine methyltransferase components of the epigenetic modification machinery. In vertebrates, it involves the post-translational modification of histone H3, at the difference of planarians instructed immunity, which relies on expression of a histone methyltransferase known to modify lysine K20 of histone H4. In term of cell actors, we show the absence of involvement of phagocytes, which contrasts with their importance in vertebrates. We rather establish the importance of stem cells thereby raising the question of the importance of this cell type in vertebrates. Interestingly, mammalian mesenchymal stem cells (MSCs) are similar to undifferentiated stem cell-like neoblats in less-evolved organisms such as planarians (Balaji et al., 2012, Labbe et al., 2012, Pittenger et al., 1999). Human MSCs possess some characteristics of immune cells, including a pro-inflammatory phenotype, an immunosuppressive phenotype, the expression of Toll-like receptor proteins and antibacterial properties that involve production of LL-37 (Bernardo and Fibbe, 2013, Krasnodembskaya et al., 2010, Tomchuck et al., 2008). However, humans stem cells-based immunity remains largely unknown due to the difficulty of investigating the biological properties of this type of cells. As planarians neoblasts, the mammalians MSCs form blastema regenerates tissues after injury. Comparative transcriptomic analysis shows the conserved pluripotency features of stem cells in planarians and mammals (Labbe et al., 2012). Thus, the conservation of the mechanism during evolution suggests that human stem cells are able to develop trained immunity, offering remarkable candidates for therapeutic usages. Indeed, trained stem cells can migrate faster and adapt to the inflamed or injured tissues, exhibit different phenotypes, as well modulate their function to establish a balance between pathogen elimination and tissue repair.

In conclusion, the current study of *S. aureus* re-infection in planarians unveils a role of neoblasts in the establishment of immune resistance to re-infection to *S. aureus* and defines the critical function of neoblasts in controlling the heightened expression of *Smed-PGRP-2* and *Smed-setd8*-*1* during re-infection. Collectively, this work supports the use of planarians as a model organism for the study of innate immune memory of bacterial infection and reveals an unexpected role of neoblasts as a central regulator of immune memory and master regulator of animal resistance to *S. aureus* infection.

## Funding Sources

This work was supported by the CNRS (PEPS 2010 to E.G.), the Scientific Cooperation Foundation “Infectiopole Sud” and the Regional Council P.A.C.A. (2009, Technical Facility to E.G.). P.A. and LL.T. are fellows of “Infectiopole Sud”. C.T. is a fellow of the French Ministry for Research and Technology and of “Fondation pour la Recherche Médicale” (FRM FDT20160435255). The funding sources had no role in study design, data collection and analysis, decision to publish, or manuscript preparation.

## Conflict of Interest Statement

The authors declare that they have no competing financial interests.

## Author Contributions

C.T., P.A., L.L.T., C.L., VT., G.G., J.D. and S.G. performed the experiments; C.T., P.A., L.L.T., G.M., G.G., L.P., E.L. and E.G. analysed the data; C.T., P.A., L.L.T., G.M., C.L., L.P., E.L., and E.G. conceived and designed the experiments; G.M., G.G., L.P., E.L., and E.G. contributed materials and reagents; and CT., G.M., E.L. and E.G. wrote the manuscript.

## Appendix A Supplementary data

Supplementary material Image 1
