# Supplementary material for: Staphylococcus aureus Promotes Smed-PGRP-2/Smed-setd8-1 Methyltransferase Signalling in Planarian Neoblasts to Sensitize Anti-bacterial Gene Responses During Re-infection
Source: eBioMedicine. 2017 Apr 24;20:150–60. doi: 10.1016/j.ebiom.2017.04.031 (PMC5478204; doi:10.1016/j.ebiom.2017.04.031)
Supplement: Supplementary file 1 — Supplementary material [file mmc1.pdf]

Supplementary data

Table S1. Expression of genes of interest in publicly available RNA sequencing raw data (GSE37910).

RNA sequencing raw data from planarian tissue and FACS-purified neoblasts published by Labbe et al. (Labbe et al., 2012) were downloaded from the GEO database (accession number: GSE37910) and analysed (see Methods section). *Smed-PGRP-2*, *Smed-setd8-1*, *Smed-morn2,* and *Smed-p38 MAP Kinase* were searched. *Smed-PGRP-2*, *Smed-Setd8-1*, *Smed-morn2,* and *Smed-p38 MAPK* expression levels in planarian tissue and sorted neoblasts are expressed in cRPKM.

Figure S1 related to Figure 1. *Smed-PGRP-2* expression drives heightened resistance in re-infection by *Staphylococcus aureus.*

(A) *S. mediterranea* were infected (primo-infection) with *S. aureus* and then re-infected forty-five days later with the same bacteria that were used for primo-infection. In this experiment, instructed immunity was not detectable. The results are expressed as the mean ± SD (five animals per time point, n=3, *p<0.05). **(B)** *Smed-PGRP-2* expression was analysed by performing RT-qPCR and was induced by *S. aureus.* The results are expressed as the mean ± SD (ten animals per time point, n=3, *p<0.05). **(C and D)** Validation of RNAi-mediated down-regulation of *Smed-PGRP-2* expression by performing RT-qPCR in **(C)** planarians and **(D)** primo-infected planarians was determined 3 days after the last RNAi treatment. *Smed-PGRP-2* mRNA expression levels decreased by 80%. The results are expressed as the mean ± SD (five animals per experimental condition, n=3). All results were analysed using the nonparametric Mann-Whitney U test. Differences were considered significant at p<0.05.

Figure S2 related to Figure 2. Neoblast-based heightened resistance to *S. aureus* re-infection.

(**A)** *Smed-CerS1* knockdown efficiency was confirmed by performing RT-qPCR three days after the last RNAi treatment. *Smed-CerS1* mRNA expression levels decreased by more than 85%. The results are expressed as the mean ± SD (five animals per experimental condition, n=3). **(B)** *Smed-H2B* knockdown efficiency was confirmed by *in situ* hybridization five days after the last RNAi treatment. *Smed-H2B* transcripts were not detectable in animals silenced for *Smed-H2B* using RNAi, unlike control animals exposed to *eGFP (RNAi)*. Representative micrographs of 3 experiments with five animals per experimental condition. Scale bars, 125 µm. **(C)** *Smed-H2B* knockdown efficiency was confirmed by performing RT-PCR three days after the last RNAi treatment. *Smed-H2B* mRNA expression decreased by 90%. The results are expressed as the mean ± SD (five animals per experimental condition, n=3, *p<0.05). **(D)** *Smedwi-3* knockdown efficiency was confirmed by performing RT-PCR three days after the last RNAi treatment. *Smedwi-3* mRNA expression decreased by 90%. The results are expressed as the mean ± SD (five animals per experimental condition, n=2, *p<0.05). **(E)** Experimental strategy used to graft instructed immunity. **(F)** Neoblasts were eliminated by irradiating at 60 Gy. Post-irradiation, *Smedwi-1* transcripts were not detectable by *in situ* hybridization five days after irradiation, unlike non-irradiated worms (control). Representative micrographs of 3 experiments with five animals per experimental condition. Scale bars, 125 µm. **(G)** Animals were irradiated at 60 Gy or left untreated; then, animals were challenged five days later with *S. aureus* for twenty-four hours. *Smed-PGRP-2* mRNA expression in planarian tissue was analysed by performing whole-mount *in situ* hybridization (representative micrographs of 2 experiments with five animals per experiment; scale bars, 125 µm). **(H)** Representative analysis of neoblasts sorted by FACS using dot plots (side scatter versus forward scatter dot plot) from 2 experiments. Cells from dissociated animals

(60 animals per experimental condition) were stained with two fluorescent dyes (Hoechst 33342 and calcein AM). Neoblasts (P2 and P3) were sorted for further experiments using a BD FACS Jazz instrument (BD Biosciences). All results were analysed using the nonparametric Mann-Whitney U test. Differences were considered significant at $p<0.05$.

Figure S3 related to Figure 3. *Smed-setd8-1* expression controls heightened resistance to *S. aureus* re-infection.

(A) Validation of RNAi-mediated silencing of histone methyltransferases was determined by performing RT-qPCR three days after the last RNAi treatment. The results are expressed as the mean ± SD (five animals per experimental condition, n=3, $^*p<0.05$). Histone methyltransferase mRNA expression levels decreased by approximately 80%. **(B)** *S. mediterranea* that were subjected to RNAi-mediated silencing of *Smed-setd8-1* were infected with *S. aureus* three days after the last RNAi treatment. Then, bacterial behaviour was monitored by CFU counting. *Smed-setd8-1* silencing did not affect the ability of planarians to eliminate *S. aureus* during primo-infection. The results are expressed as the mean ± SD (five animals per time point, n=3). **(C)** Whole-mount *in situ* hybridization analysis of *Smed-setd8-1* expression in control and *S. aureus*-infected animals fixed at thirty-six hours post-infection (representative micrographs of 2 experiments with five animals per experimental condition; scale bars, 125 µm). **(D)** *Smed-setd8-1* expression in planarians depleted of neoblasts by 60 Gy irradiation was determined by performing RT-qPCR five days after irradiation. The results are expressed as the mean ± SD (five animals per experimental condition, n=3, $^*p<0.05$). **(E)** Representative FACS dot plots of 4 experiments using dissociated non-irradiated (control) and four-day planarians post-60-Gy irradiation. Cells from dissociated animals were stained with two fluorescent dyes and analysed for their fluorescent intensities using a cytometer. Neoblasts are indicated with X1 (right panel); these cells were eliminated following X-ray-

irradiation (X1, left panel). (**F)** Representative cytogram from 3 experiments. Lysine methylation levels in neoblast population X1 isolated from planarians silenced for *Smed-setd8-1* and challenged with *S. aureus* for thirty-six hours. All results were analysed using the nonparametric Mann-Whitney U test. Differences were considered significant at p<0.05.

Figure S4 related to Figure 4. Smed-PGRP-2 promotes *Smed-setd8-1*-dependent induction of anti-microbial gene responses.

(A, B) *Smed-p38* MAP kinase **(A)** and *Smed-morn2* mRNA levels **(B)** were determined by performing RT-qPCR in animals that were infected with *L. pneumophila* and then re-infected thirty days later with *L. pneumophila.* **(A)** *Smed-p38* MAP kinase and **(B)** *Smed-morn2* mRNA were similarly expressed during *L. pneumophila* infection and infection recall. The results are expressed as the mean ± SD (five animals per time point, n=2, *p<0.05). **(F)** *Smed-PGRP-2* mRNA levels in animals silenced for *Smed-Setd8-1* and then challenged with *S. aureus* for twenty-four hours were evaluated by performing RT-qPCR. *Smed-PGRP-2* mRNA expression in *S. aureus*-infected worms was unaffected by *Smed-PGRP-2* knockdown. The results are expressed as the mean ± SD (five animals per experimental condition, n=3, *p<0.05). All results were analysed using the nonparametric Mann-Whitney U test. Differences were considered significant at p<0.05.

Table S1. Expression of genes of interest in publicly available RNA sequencing raw data (GSE37910).

genes name	cRPKM Tissue	cRPKM Neoblast
Smed-PGRP-2	92.72	1.71
Smed-Setd8-1	3.97	65.8
Smed-morn2	12.67	16.39
Smed-p38 MAPK	93.95	27.5

figure S1

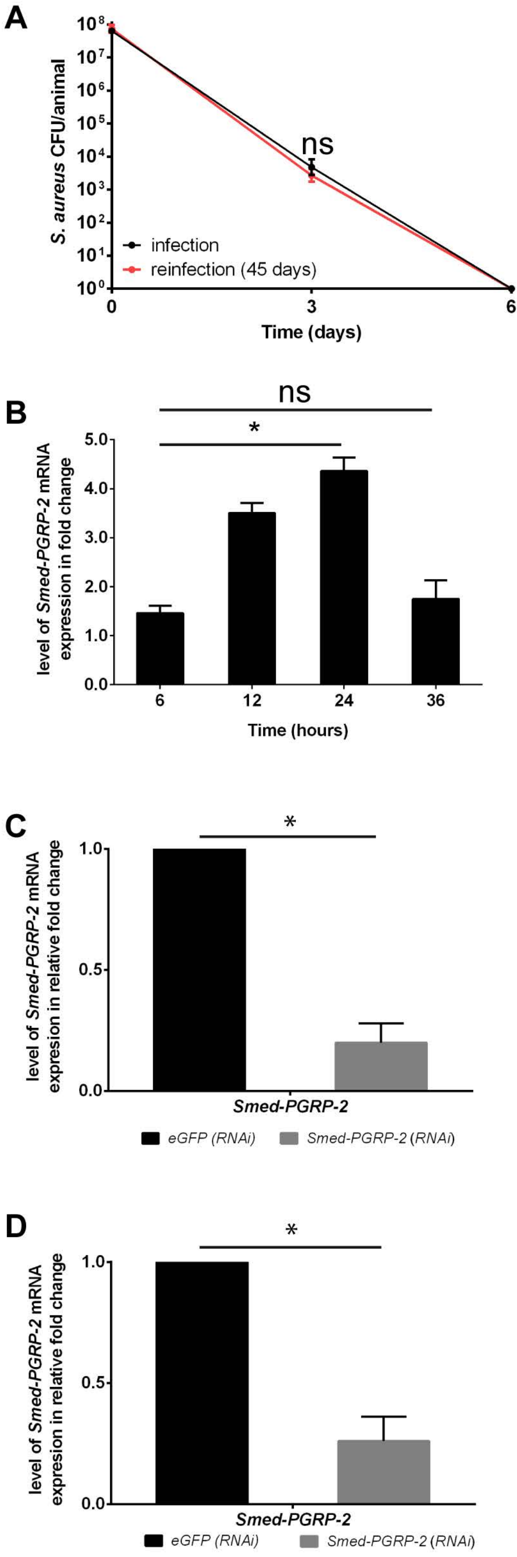
A
10^8
10^7
10^6
10^5
10^4
10^3
10^2
10^1
10^0
S. aureus CFU/animal
ns
infection
reinfection (45 days)
0
3
6
Time (days)
B
ns
*
5.0
4.0
3.0
2.0
1.0
0.0
level of Smed-PGRP-2 mRNA expression in fold change
6
12
24
36
Time (hours)
C
*
1.0
0.5
0.0
level of Smed-PGRP-2 mRNA expresion in relative fold change
Smed-PGRP-2
eGFP (RNAi)
Smed-PGRP-2 (RNAi)
D
*
1.0
0.5
0.0
level of Smed-PGRP-2 mRNA expresion in relative fold change
Smed-PGRP-2
eGFP (RNAi)
Smed-PGRP-2 (RNAi)

figure S2

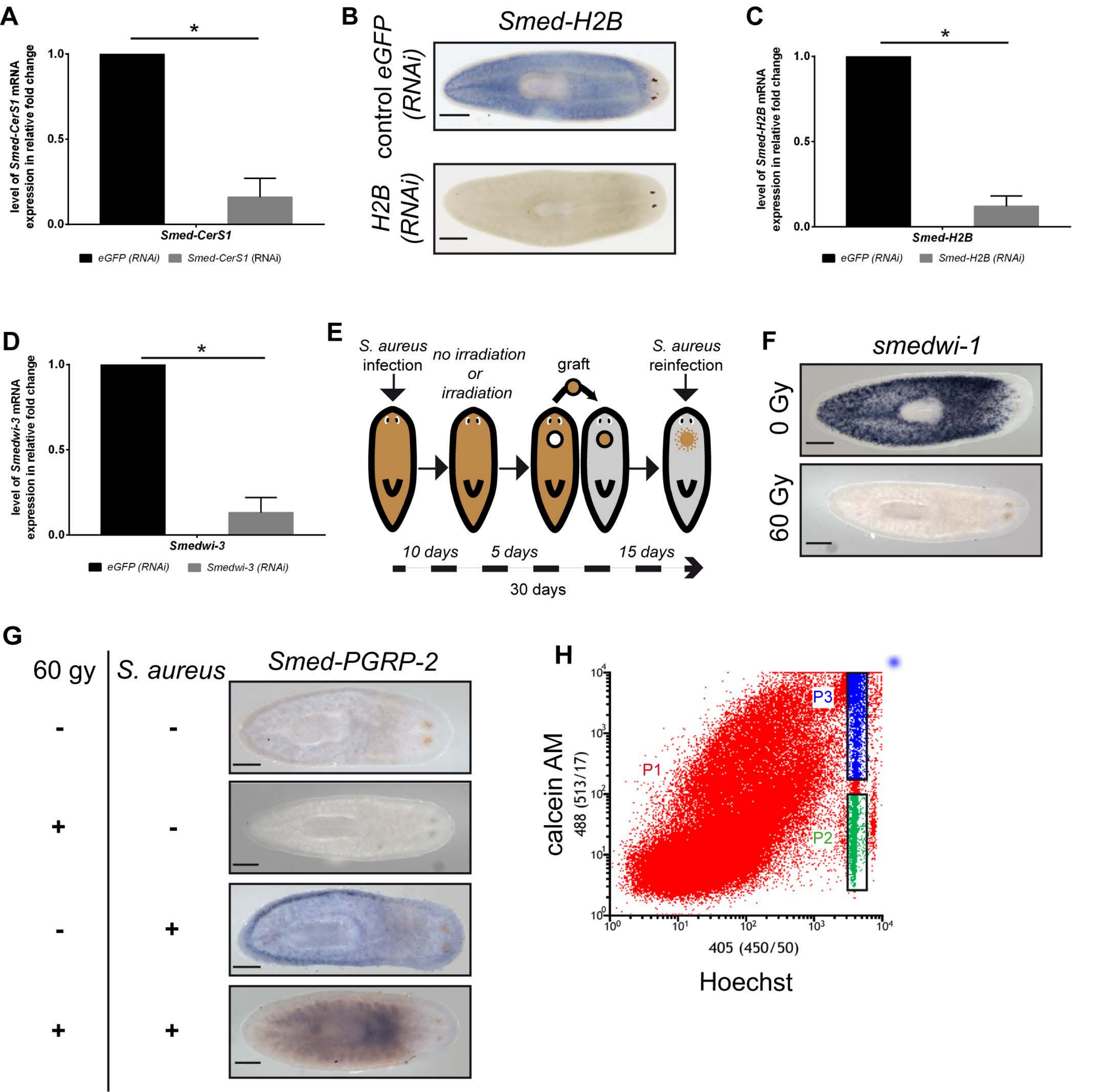

A
*
1.0
0.5
0.0
level of Smed-CerS1 mRNA expression in relative fold change
Smed-CerS1
eGFP (RNAi)
Smed-CerS1 (RNAi)
B
Smed-H2B
control eGFP (RNAi)
H2B (RNAi)
C
*
1.0
0.5
0.0
level of Smed-H2B mRNA expression in relative fold change
Smed-H2B
eGFP (RNAi)
Smed-H2B (RNAi)
D
*
1.0
0.5
0.0
level of Smedwi-3 mRNA expression in relative fold change
Smedwi-3
eGFP (RNAi)
Smedwi-3 (RNAi)
E
S. aureus infection
no irradiation or irradiation
graft
S. aureus reinfection
10 days
5 days
15 days
30 days
F
smedwi-1
0 Gy
60 Gy
G
60 gy
S. aureus
Smed-PGRP-2
-
-
+
-
-
+
+
+
H
P1
P2
P3
calcein AM
488 (513/17)
405 (450/50)
Hoechst

figure S3

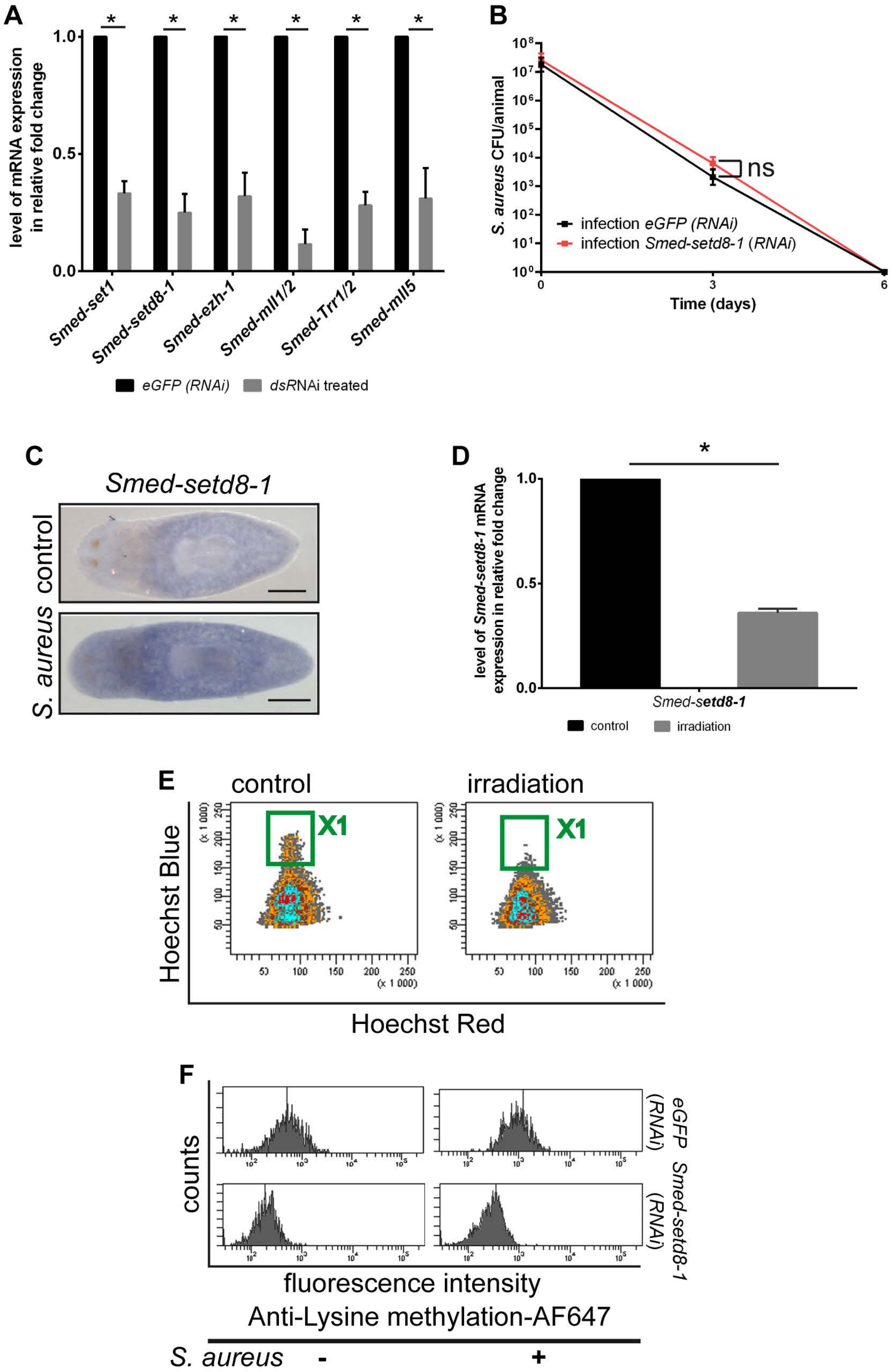
A
level of mRNA expression in relative fold change
1.0
0.5
0.0
Smed-set1
Smed-setd8-1
Smed-ezh-1
Smed-mll1/2
Smed-Trr1/2
Smed-mll5
eGFP (RNAi)
dsRNAi treated
B
S. aureus CFU/animal
ns
infection eGFP (RNAi)
infection Smed-setd8-1 (RNAi)
Time (days)
0
3
6
C
Smed-setd8-1
control
S. aureus
D
level of Smed-setd8-1 mRNA expression in relative fold change
1.0
0.5
0.0
Smed-setd8-1
control
irradiation
E
control
irradiation
X1
X1
Hoechst Blue
Hoechst Red
F
counts
eGFP (RNAi)
Smed-setd8-1 (RNAi)
fluorescence intensity
Anti-Lysine methylation-AF647
S. aureus
-
+

figure S4

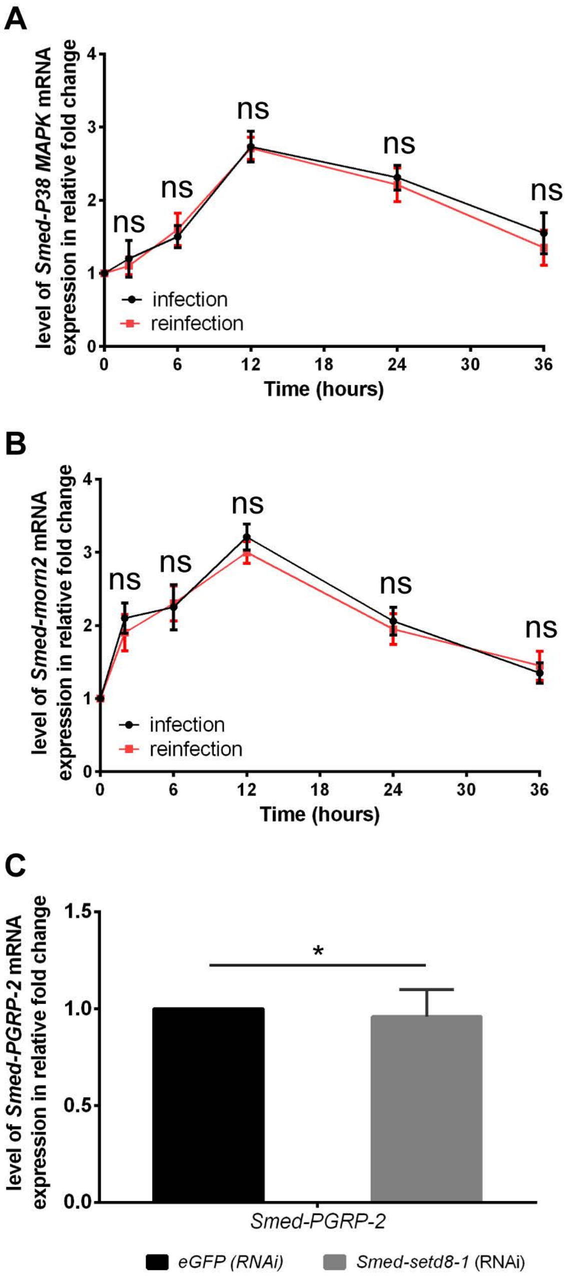
A
level of Smed-P38 MAPK mRNA expression in relative fold change
ns
ns
ns
ns
ns
infection
reinfection
Time (hours)
0 6 12 18 24 30 36
0 1 2 3 4
B
level of Smed-morn2 mRNA expression in relative fold change
ns
ns
ns
ns
ns
infection
reinfection
Time (hours)
0 6 12 18 24 30 36
0 1 2 3 4
C
level of Smed-PGRP-2 mRNA expression in relative fold change
*
0.0 0.5 1.0 1.5
Smed-PGRP-2
eGFP (RNAi)
Smed-setd8-1 (RNAi)
